# Association between Sarcopenia and Physical Function among Preoperative Lung Cancer Patients

**DOI:** 10.3390/jpm10040166

**Published:** 2020-10-13

**Authors:** Sunga Kong, Sumin Shin, Jae Kyung Lee, Genehee Lee, Danbee Kang, Juhee Cho, Hong Kwan Kim, Jae Ill Zo, Young Mog Shim, Hye Yun Park, Dong Wook Shin

**Affiliations:** 1 Department of Clinical Research Design and Evaluation, SAIHST, Sungkyunkwan University, Seoul 06351, Korea; sunga00kong@gmail.com (S.K.); genehee@gmail.com (G.L.); dbee.kang@gmail.com (D.K.); jcho@skku.edu (J.C.); 2 Patient-Centered Outcomes Research Institute, Samsung Medical Center, Seoul 06351, Korea; roemgirls1230@gmail.com; 3 Department of Thoracic and Cardiovascular Surgery, Samsung Medical Center, School of Medicine, Sungkyunkwan University, Seoul 06351, Korea; essennee02@gmail.com (S.S.); hkts.kim@samsung.com (H.K.K.); jayl.zo@samsung.com (J.I.Z.); youngmog.shim@samsung.com (Y.M.S.); 4 Center for Clinical Epidemiology, Samsung Medical Center, School of Medicine, Sungkyunkwan University, Seoul 06351, Korea; 5 Departments of Epidemiology and Health, Behavior and Society, Johns Hopkins Bloomberg School of Public Health, Baltimore, MD 21205, USA; 6 Division of Pulmonary and Critical Care Medicine, Department of Medicine, Samsung Medical Center, School of Medicine, Sungkyunkwan University, Seoul 06351, Korea; 7 Department of Family Medicine & Supportive Care Center, Samsung Medical Center, Seoul 06351, Korea; 8 Department of Digital Health, SAIHST, Sungkyunkwan University, Seoul 06351, Korea

**Keywords:** sarcopenia, lung cancer, skeletal muscle mass, muscle strength, physical activity, cardiopulmonary function, preoperative

## Abstract

We aimed to investigate the prevalence of sarcopenia using new diagnostic criteria and association of sarcopenia with cardiopulmonary function and physical activity (PA) in preoperative lung cancer patients. The data of 614 patients were obtained from the CATCH-LUNG cohort study. Patients were classified into three groups—normal (*n* = 520), pre-sarcopenia (*n* = 60, low skeletal muscle mass index only), and sarcopenia (*n* = 34, low SMI and strength). Cardiopulmonary function was measured using the 6-min walk test (6MWT), and PA was objectively measured using a wearable device. The adjusted odds ratio (aOR) for a <400-m distance in 6MWT was 3.52 (95% confidence interval (CI) 1.34–9.21) and 6.63 (95% CI 2.25–19.60) in the pre-sarcopenia and sarcopenia groups, respectively, compared to that in the normal group. The aOR (95% CI) for <5000 steps/day was 1.64 (0.65–4.16) and 4.20 (1.55–11.38) in the pre-sarcopenia and sarcopenia groups, respectively, compared to that in the normal group. In conclusion, the prevalence of pre-sarcopenia and sarcopenia was 9.8% and 5.5%, respectively, among preoperative lung cancer patients. Cardiopulmonary function and physical activity were significantly lower in the pre-sarcopenia and sarcopenia groups than in the normal group. Patients with sarcopenia had more robust findings, suggesting the importance of muscle strength and mass.

## 1. Introduction

The median age at diagnosis of lung cancer is 70 years [1], and more than 70% of future lung cancer cases are expected to occur in adults older than 65 years [2]. With the increasing number of elderly patients with lung cancer, sarcopenia in non-small cell lung cancer (NSCLC) has received attention. The prevalence of sarcopenia varies depending on the diagnostic criteria used to define the condition; however, it is up to 43% in NSCLC [3].

Sarcopenia was previously defined as loss of skeletal muscle mass with aging [4]. As loss of muscle function is more relevant to clinical outcomes than low muscle mass only, the importance of generalized muscle function has been underscored and muscle strength is now included as an additional parameter to low muscle mass for the diagnosis of sarcopenia in the European Working Group on Sarcopenia in Older People and the Asian Working Group for Sarcopenia (AWGS) guidelines [5,6].

Surgical resection remains the best treatment option in patients with early-stage NSCLC. Cardiopulmonary function assessment is a major process in determining the feasibility of surgical resection as poor preoperative cardiopulmonary function and physical activity are associated with perioperative complications [7,8]. Further, the presence of sarcopenia, defined as low muscle mass only, has been associated with a greater risk of perioperative complications and worse long-term outcomes after lung cancer surgery [9]. Sarcopenia is also related to cancer-related malnutrition having poor nutrition intake and weight loss, which is considered as a prognostic factor in NSCLC [10,11]. However, there is no recommendation to assess the presence of sarcopenia and no available study documenting the association of sarcopenia with cardiopulmonary function and physical activity in preoperative lung cancer patients. Given that sarcopenia can be improved by preoperative rehabilitation [12], investigating this association is important. Thus, we aimed to investigate the prevalence of sarcopenia using the new diagnostic criteria and the association between sarcopenia and cardiopulmonary function and physical activity in preoperative lung cancer patients.

## 2. Methods

### 2.1. Study Participants

We used baseline data from the Coordinate Approach to Cancer Patients’ Health for Lung Cancer (CATCH-LUNG) prospective cohort study (Clinical Trials.gov ID: NCT03705546). Patients in the CATCH-LUNG study were recruited from a tertiary university-based cancer center from March 2016 to October 2018 in Seoul, South Korea [13]. Patients who met the inclusion criteria were those (1) who were expected to undergo curative lung cancer surgery for suspected or histologically confirmed NSCLC, (2) whose Eastern Cooperative Oncology Group Performance Status (ECOG-PS) was either 0 or 1, (3) who had no problems with walking, and (4) who understood the purpose of the study and agreed to participate. Patients were excluded if (1) they had undergone neoadjuvant treatment before surgery, (2) they were confirmed to have no NSCLC on pathologic examinations, (3) their surgery was canceled, or (4) they withdrew consent before baseline data collection. Among 620 patients who met these criteria, analyses were limited to patients who had grip strength or skeletal muscle mass measurements for this study. Finally, 614 patients were included for the analysis. The study protocol was approved by the Institutional Review Board of Samsung Medical Center (approval no. 2015-11-025). Written informed consent was obtained from all participants.

### 2.2. Muscle Mass and Hand Grip Strength for Sarcopenia Group

The participants were classified into three groups: (1) normal; (2) pre-sarcopenia, defined as low muscle mass only without impairment of muscle strength [14]; and (3) sarcopenia, defined as both low muscle mass and impaired muscle strength [6].

Muscle mass estimated body composition, including height, weight, body fat, and appendicular skeletal muscle mass (ASM), was measured using InBody 770 (InBody Co., Ltd., Seoul, Korea). Skeletal muscle mass index (SMI) for muscle mass assessment was calculated as the sum of the ASM of the arms and legs divided by height in meters squared (kg/m^2^) [15]. The accuracy of bioelectrical impedance analysis in sarcopenia diagnosis was validated [16].

Hand grip strength was recorded as the measure for muscle strength. The mean hand grip strength (0.1 kg) was calculated from the total of four measurements (two with the left hand and two with the right hand) taken using a digital dynamometer (T.K.K.5401; Takei Scientific Instruments Co., Ltd., Niigata, Japan).

The cutoff values for muscle mass and muscle strength were determined according to the AWGS recommendations (cutoff values of skeletal muscle mass index for muscle mass assessment using bioelectrical impedance analysis: <7.0 kg/m^2^ for men and <5.7 kg/m^2^ for women; cutoff values of hand grip strength for muscle strength: <28 kg for men and <18 kg for women) [6].

### 2.3. Cardiopulmonary Function and Physical Activity

Cardiopulmonary function was measured using the 6-min walk test (6MWT) according to the American Thoracic Society guidelines [17]. Each participant was asked to walk (not run) back and forth along the corridor as far as possible for 6 min and given standardized verbal encouragement every minute. The low cardiopulmonary function cutoff point was defined as a <500 m distance [18] or a <400 m distance [5,7,19]

Physical activity was objectively measured using Flex (Fitbit, San Francisco, CA, USA) [20]. The Flex tracker was used to quantify the physical activity intensity (light, moderate, or vigorous), activity time by intensity (minutes per day), and steps per day. The tracker was used for 7 consecutive days to measure the physical activity level during both weekdays and weekends before surgery for NSCLC. The tracker was worn on the wrist. To reduce bias, the device had no screen; hence, the patients could not see their recorded level of physical activity [13]. Being physically inactive was defined as either taking <5000 steps/day [21,22], or performing <30 min of moderate-to-vigorous physical activity (MVPA) per day [23]. 

### 2.4. Covariates

Spirometry and diffusing lung capacity of carbon monoxide (DLco) measurements were performed using Vmax 22 (SensorMedics, Yorba Linda, CA, USA) according to the American Thoracic Society/European Respiratory Society criteria [24,25]. Absolute values of forced expiratory volume in 1 s (FEV_1_), forced vital capacity (FVC), and DLco were obtained, and the percentages of predicted values for FEV_1_, FVC, and DLco were calculated using a reference equation obtained on analysis of a representative South Korean sample, which adjusted for the standard hemoglobin level [26,27]. 

Information on marital status, education level, smoking status, and comorbidities was obtained using questionnaires. Comorbidity consisted of pulmonary comorbidities including chronic obstructive pulmonary disease (COPD), asthma, interstitial lung disease, and extrapulmonary comorbidities such as diabetes mellitus, hypertension, and cardiovascular disease. Laboratory findings including serum hemoglobin and albumin levels were obtained from electronic medical records.

### 2.5. Statistical Analyses

Descriptive statistics were used to summarize the characteristics of participants between groups (normal, pre-sarcopenia, and sarcopenia groups). Continuous and categorical variables were compared between groups using analysis of variance (ANOVA) with post-hoc Scheffé test and χ^2^ test, respectively. 

Linear regression analysis was performed to compare the adjusted mean and 95% confidence interval (CI) of 6MWT distance, steps per day, and the time of physical activity level between groups, after adjusting for age sex, body mass index (BMI), and FEV_1_ <50% of the predicted value. Logistic regression analysis was performed to compare the odds of low cardiopulmonary function and low physical activity after adjusting for age, sex, BMI, and FEV_1_ <50% of the predicted value. We developed a dichotomized outcome to evaluate the proportion of participants who had low cardiopulmonary function and were physically inactive for each group. 

We considered a *p* value and *p* for trend value of <0.05 as statistically significant for the comparison of the normal, pre-sarcopenia, and sarcopenia groups. All analyses were performed using STATA version 15 (Stata Corp LP, College Station, TX, USA).

## 3. Results

### 3.1. Patient Characteristics

The patient characteristics are described in Table 1. The mean (standard deviation) age of the patients was 61.2 (9.0) years, and 56.5% (*n* = 347) of the total patients were men. Of the 614 patients, 520 (84.7%) were in the normal group, 60 (9.8%) were in the pre-sarcopenia group, and 34 (5.5%) were in the sarcopenia group. Patients in the sarcopenia group were older and more likely to have lower BMI, pulmonary function, and albumin and hemoglobin levels than those in the normal and pre-sarcopenia groups (*p* < 0.001). However, there was no significant difference in terms of smoking history, education level, marital status, pathologic stage, and comorbidities across the groups, except for a higher prevalence of COPD in the sarcopenia group.

### 3.2. Cardiopulmonary Function by Sarcopenia Groups

The difference in the cardiopulmonary function of each group is shown in Table 2.

The distance in the 6MWT gradually decreased across the groups; the adjusted mean (95% confidence interval [CI]) distance in each group was 519.5 (512.9–526.2), 487.9 (467.7–508.2), and 464.5 (436.9–492.1) m, respectively (*p* for trend <0.001).

**Figure 1:**
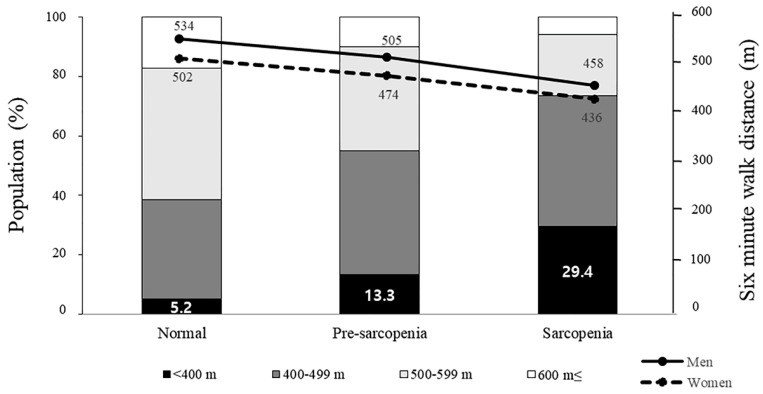
Six-minute walking distance according to sarcopenia group.

The proportion of patients with 6MWT distance <500 m was 38.5%, 55.0%, and 73.5% in the normal, pre-sarcopenia, and sarcopenia groups, respectively (*p* for trend <0.001), and the adjusted odds ratios (aORs) and 95% CIs for the pre-sarcopenia and sarcopenia groups were 2.00 (1.09–3.68) and 3.55 (1.48–8.51), respectively, compared to those for the normal group (*p* for trend = 0.001) (Figure 1). The proportion of patients with 6MWT distance < 400 m was 5.2%, 13.3%, and 29.4% in the normal, pre-sarcopenia, sarcopenia groups, respectively, and the aORs (95% CIs) for the pre-sarcopenia and sarcopenia groups were 3.52 (1.34–9.21) and 6.63 (2.25–19.60), respectively, compared to those for the normal group (*p* for trend < 0.001).

When we conducted additional analysis using sex, the results were similar (Supplementary Table S1).

### 3.3. Physical Activity by Sarcopenia Groups

Of the 552 patients with available Fitbit data analysis, 475 (86.0%) were in the normal group, 49 (8.9%) were in the pre-sarcopenia group, and 28 (5.1%) were in the sarcopenia group. The difference in physical activity between each group is shown in Table 3. The adjusted mean (95% CI) steps per day in each group was 10,970 (10,499–11,440); in normal group, 8785 (7270–10,300); in pre-sarcopenia group; and 7160 (5124–9196) in sarcopenia group (*p* for trend <0.001). The aOR (95% CI) for <5000 steps/day was 1.64 (0.65–4.16) in the pre-sarcopenia group and 4.20 (1.55–11.38) in the sarcopenia group with reference of the normal group (*p* for trend = 0.006). In addition, patients in the pre-sarcopenia and sarcopenia groups were more likely to have less time for MVPA than patients in the normal group. The aOR (95% CI) for MVPA <30 min/day was 1.93 (1.02–3.67) in pre-sarcopenia group and 2.86 (1.22–6.69) in the sarcopenia group compared to that in the normal group (*p* for trend = 0.004). 

## 4. Discussion

In this study, new diagnostic criteria for sarcopenia were used for the first time to assess the relationship with cardiopulmonary function and physical activity in patients before surgical resection of NSCLC. The prevalence of pre-sarcopenia and sarcopenia was 9.8% and 5.5%, respectively, in our study population. Furthermore, we found that NSCLC patients with preoperative sarcopenia were more likely to have a shorter distance in the 6MWT and less physical activity compared to those of NSCLC patients in the normal group, and these results were more robust among patients with sarcopenia than in those with pre-sarcopenia.

To date, no study has directly reported the association between sarcopenia and cardiopulmonary function in patients who are scheduled to undergo surgical resection for NSCLC. Some previous studies have reported such an association in different populations, such as community-dwelling older people or patients with heart failure [28,29,30,31]. Of these, one study involving patients with heart failure reported that low skeletal muscle mass was linked to reduced exercise capacity, and patients with sarcopenia recorded less distance than patients without sarcopenia (307 ± 145 vs. 404 ± 116 m) in 6MWT [32]. Another study showed that muscle strength was associated with impaired functional performance (chair stand test and usual and fast gait speed) among older adults [29]. Our study extended these results with new criteria of sarcopenia and demonstrated that sarcopenia, defined using both muscle strength and mass, has a stronger relationship to cardiopulmonary function than pre-sarcopenia, defined using muscle mass alone. 6MWT has been recognized as an assessment tool for functional exercise capacity in patients with lung cancer [33], and patients with a shorter distance of <400 or <500 m would have an additional risk for postoperative complication [7,18]. Therefore, our findings suggest that more attention to identify low exercise capacity should be paid to sarcopenia patients with both low skeletal mass and low muscle strength, more than patients with only low skeletal mass. 

In terms of daily physical activity, our study showed that the presence of sarcopenia was correlated with less physical activity time and fewer step counts in our study. This is consistent with previous studies reporting a relationship between sarcopenia and reduced daily step counts [34] and longer sedentary time [35]. One previous study in patients with COPD found that the sarcopenia group with low muscle function only showed significant reduction in self-reported physical activities compared to the sarcopenia with low muscle mass only group and normal group; however, there was no significant difference in the self-reported physical activities between the normal group and the group with sarcopenia, defined as low muscle mass only [36]. Our results objectively showed that reduced physical activity was more pronounced in the sarcopenia group than in the pre-sarcopenia group, suggesting the importance of muscle strength. 

In our study, patients with sarcopenia had poorer cardiopulmonary function and less physical activity than those with pre-sarcopenia. Although muscle mass and muscle strength are closely related, muscle strength is more predictive of the adverse outcomes of sarcopenia than muscle mass [37]. Therefore, muscle strength is prioritized in the definition and assessment of sarcopenia in the current guidelines [5,6]. However, previous studies investigating sarcopenia in surgical patients [12,38,39,40,41], including patients with NSCLC [3,42], have used muscle mass only in their evaluation of sarcopenia. Our findings indicate that assessment of low muscle strength needs to be considered a central component in the assessment of sarcopenia in patients who are scheduled for lung cancer surgery, which is consistent with the current sarcopenia guidelines [5,6].

Our study also suggests the potential benefit of an intervention for reducing preoperative sarcopenia. Preoperative rehabilitation to enhance physical activity (both resistance and aerobic exercise) and nutritional support (combination with adequate protein and energy intake) can improve muscle mass and strength before surgery for NSCLC [43,44]. In addition, there is substantial evidence to link nutrition to muscle mass and strength and weight loss is common with the diagnosis of lung cancer, which, in turn, is related to muscle loss [45]. Thus, a comprehensive preoperative intervention, including adequate rehabilitation program and nutritional support, would be necessary to improve muscle strength and physical function in patients who are scheduled to undergo surgical resection for lung cancer.

Our study had several limitations. First, as this study was conducted at a tertiary hospital in Seoul, South Korea, the results might not be generalizable to different settings. Second, as our cohort only included patients with ECOG-PS of 0 or 1, they would be relatively healthier patients and more physically active than general lung cancer patients. Therefore, the prevalence of sarcopenia was lower in this study than that reported in other studies, and the clinical significance of sarcopenia can be underestimated among patients with good performance status. Finally, CATCH-LUNG was not originally designed to identify the presence of sarcopenia among preoperative lung cancer patients, and the analysis (by definition) had to be retrospective in nature.

## 5. Conclusions

In this study, we found that the prevalence of pre-sarcopenia and sarcopenia were 9.8% and 5.5%, respectively, among preoperative lung cancer patients. In addition, cardiopulmonary function, measured using 6MWT, and physical activity level measured by a wearable device, were significantly lower in the pre-sarcopenia and sarcopenia groups than in the normal group. These findings were more robust in patients with sarcopenia than pre-sarcopenia group, suggesting the importance of muscle strength in addition to muscle mass.

## Figures and Tables

**Table 1:** Characteristics of study participants by sarcopenia group (*n* = 614).

Characteristics	Normal (*n* = 520)	Pre-Sarcopenia (*n* = 60)	Sarcopenia (*n* = 34)	*p*
**Age, years**	60.4 (8.6)	63.0 (8.9)	70.2 (10.4) ^a,b^	<0.001
**Men, sex**	305 (58.7)	26 (43.3)	16 (47.1)	0.040
**Body mass index, kg/m^2^**	24.7 (2.6)	21.4 (2.3) ^a^	21.0 (2.9) ^a^	<0.001
Smoking				0.162
Never smoker	255 (49.0)	30 (50.0)	11 (32.4)	
Ever smoker	265 (51.0)	30 (50.0)	23 (67.6)	
**Education level**				0.262
<High school	319 (61.4)	42 (70.0)	24 (70.6)	
≥High school	201 (38.6)	18 (30.0)	10 (29.4)	
**Marital status**				0.084
Married	465 (89.4)	48 (80.0)	31 (91.2)	
Unmarried	55 (10.6)	12 (20.0)	3 (8.8)	
Comorbidities				
**Pulmonary comorbidities**				
^#^ COPD	107 (20.6)	18 (30.0)	15 (44.1)	0.002
Asthma	10 (1.9)	3 (5.0)	1 (2.9)	0.308
ILD	1 (0.2)	0 (0.0)	0 (0.0)	0.913
**Extrapulmonary comorbidities**				
Hypertension	180 (34.6)	17 (28.3)	14 (41.2)	0.431
Diabetes mellitus	74 (14.2)	11 (18.3)	7 (20.6)	0.450
Cardiovascular disease	17 (3.3)	1 (1.7)	2 (5.9)	0.542
**Laboratory findings**				
Albumin, g/dL	4.5 (0.3)	4.4 (0.3) ^a^	4.3 (0.3) ^a^	<0.001
Hemoglobin, g/dL	13.8 (1.4)	13.4 (1.1)	12.6 (1.4) ^a,b^	<0.001
**Pathologic stage**				0.326
I	378 (72.7)	47 (78.3)	22 (64.7)	
II	82 (15.8)	6 (10.0)	7 (20.6)	
III	58 (11.2)	7 (11.7)	4 (11.8)	
IV	2 (0.4)	0 (0.00)	1 (2.9)	
**Pulmonary function**				
FVC, L	3.7 (0.8)	3.1 (0.7) ^a^	2.8 (0.6) ^a^	<0.001
FVC, % predicted	93.6 (12.3)	90.8 (12.7)	87.1 (14.1) ^a^	0.005
FEV_1_, L	2.7 (0.6)	2.3 (0.5) ^a^	1.9 (0.6) ^a,b^	<0.001
FEV_1_, % predicted	91.2 (13.6)	85.8 (17.3) ^a^	81.6 (19.0) ^a^	<0.001
FEV_1_/FVC	74.2 (7.8)	72.8 (11.2)	67.8 (12.6) ^a,b^	<0.001
DLco, % predicted	91.1 (15.3)	84.5 (16.5) ^a^	79.6 (16.6) ^a^	<0.001
**Factors of Sarcopenia definition**				
SMI (kg/m^2^)	7.41 (0.89)	5.94 (0.71) ^a^	5.80 (0.74) ^a^	<0.001
HGS (kg)	32.01 (8.60)	26.80 (6.64) ^a^	20.47 (4.76) ^a,b^	<0.001

Values are number of patients (%) or mean (standard deviation). Pre-sarcopenia: pre-sarcopenia group defined according to skeletal muscle mass (SMI < 7.0kg/m^2^ (Men) or 5.7kg/m^2^ (Women)). Sarcopenia: sarcopenia group defined according to both skeletal muscle mass (SMI < 7.0 kg/m^2^ (Men) or 5.7 kg/m^2^ (Women)) and hand grip strength (HGS >28kg (Men) or 18kg (Women)). ^#^ COPD is defined as a pre-bronchodilator FEV1/FVC < 70%. ^a^ *p* < 0.05 versus normal group. ^b^ *p* < 0.05 versus the pre-sarcopenia group. COPD, chronic obstructive pulmonary disease; ILD, interstitial lung disease; FVC, forced vital capacity; FEV_1_, forced expiratory volume in 1 s; DLco, diffusing capacity of carbon monoxide of the lung. SMI, skeletal muscle mass. HGS, hand grip strength.

**Table 2:** Differences in preoperative cardiopulmonary function by sarcopenia status.

	Normal (*n* = 520)	Pre-Sarcopenia (*n* = 60)	Sarcopenia (*n* = 34)	*p* for Trend
**6MWT (m)**				
6MWD (95% CI)	519.5 (512.9–526.2)	487.9 (467.7–508.2) ^a^	464.5 (436.9–492.1) ^a^	<0.001
**Categorical variable**				
**6MWD < 500 m (*n*, %)**	200 (38.5)	33 (55.0)	25 (73.5)	<0.001
OR (95% CI)	Reference	1.96 (1.14–3.35) ^a^	4.44 (2.03–9.72) ^a^	<0.001
aOR (95% CI)	Reference	2.00 (1.09–3.68) ^a^	3.55 (1.48–8.51) ^a^	0.001
**6MWD < 400 m (*n*, %)**	27 (5.2)	8 (13.3)	10 (29.4)	<0.001
OR (95% CI)	Reference	2.81 (1.21–6.50) ^a^	7.61 (3.31–17.50) ^a^	<0.001
aOR (95% CI)	Reference	3.52 (1.34–9.21) ^a^	6.63 (2.25–19.60) ^a^	<0.001

Values of 6MWD are presented as means and 95% confidence intervals adjusted for age, sex, body mass index, and forced expiratory volume in 1s (FEV_1_) < 50% of predicted. Pre-sarcopenia: pre-sarcopenia group defined according to skeletal muscle mass. Sarcopenia: sarcopenia group defined according to both skeletal muscle mass and hand grip strength. 6MWT, 6-min walk test; 6MWD, 6-min walk distance; OR, odds ratio; CI, confidence interval; aOR, adjusted odds ratio (OR was adjusted for age sex, body mass index, and FEV_1_ < 50% of predicted). ^a^ *p* < 0.05 compared with the normal group.

**Table 3:** Differences of preoperative physical activity measured using Fitbit by sarcopenia status.

	Normal (*n* = 475)	Pre-Sarcopenia (*n* = 49)	Sarcopenia (*n* = 28)	*p* for Trend
**Physical activity (95% CI)**				
**Steps per day**	10,970 (10,499–11,440)	8785 (7270–10,300) ^a^	7160 (5124–9196) ^a^	<0.001
Light activity time	244.2 (235.4–253.0)	228.1 (199.7–256.6)	196.3 (158.0–234.5) ^a^	0.017
Moderate activity time	30.7 (27.5–33.5)	19.8 (10.9–28.7) ^a^	11.3 (−0.6–23.2) ^a^	<0.001
Vigorous activity time	25.6 (23.4–27.9)	16.5 (9.2–23.8) ^a^	12.4 (2.6–22.2) ^a^	0.002
**Categorical variable**				
**Steps < 5000/day (*n*, %)**	49 (10.3)	7 (14.3)	9 (32.1)	0.005
OR (95% CI)	Reference	1.45 (0.62–3.40)	4.12 (1.77–9.60) ^a^	0.002
aOR (95% CI)	Reference	1.64 (0.65–4.16)	4.20 (1.55–11.38) ^a^	0.006
**MVPA < 30 min/day (*n*, %)**	162 (34.1)	23 (46.9)	15 (53.6)	0.030
OR (95% CI)	Reference	1.71 (0.94–3.09)	2.23 (1.04–4.80) ^a^	0.010
aOR (95% CI)	Reference	1.93 (1.02–3.67) ^a^	2.86 (1.22–6.69) ^a^	0.004

Values of physical activity time are presented as means and 95% confidence intervals adjusted for age, sex, body mass index, and forced expiratory volume in 1 s (FEV_1_) < 50% of predicted. Pre-sarcopenia: pre-sarcopenia group defined according to skeletal muscle mass. Sarcopenia: sarcopenia group defined according to both skeletal muscle mass and hand grip strength. OR, odds ratio; CI, confidence interval; MVPA, moderate-to-vigorous physical activity; aOR, adjusted odds ratio (OR was adjusted for age sex, body mass index, and FEV_1_ < 50% of predicted). ^a^ *p* < 0.05 compared with the normal group.

## Supplementary Materials

The following are available online at https://www.mdpi.com/2075-4426/10/4/166/s1.
