# Supplementary material for: Association between Sarcopenia and Physical Function among Preoperative Lung Cancer Patients
Source: J Pers Med. 2020 Oct 13;10(4):166. doi: 10.3390/jpm10040166 (PMC7712435; doi:10.3390/jpm10040166)
Supplement: Supplementary file 1 [file jpm-10-00166-s001.pdf]

Supplement Table 1. Differences in preoperative cardiopulmonary function by sarcopenia status and sex

	Normal	Pre-sarcopenia	Sarcopenia	*p* for trend
**Men**	**(*n* = 305)**	**(*n* = 26)**	**(*n* = 16)**	
6MWD (95% CI)	542.02(537.65-546.39)	516.52 (503.44-529.59)[a]	475.94 (453.31-498.58)[a]	0.008
**Categorical variable**				
**6MWD <500 m (n, %)**	99 (32.46)	12 (46.15)	10 (62.50)	0.025
OR (95% CI)	Reference	1.78 (0.80-4.00)	3.47 (1.23-9.81)[a]	0.009
aOR (95% CI)	Reference	1.78 (0.71-4.17)	2.00 (0.61-6.57)	0.148
**6MWD <400 m (n, %)**	13 (4.26)	3 (11.54)	3 (18.75)	<0.001
OR (95% CI)	Reference	2.93 (0.78-11.02)	5.18 (1.31-20.46)[a]	0.008
aOR (95% CI)	Reference	3.90 (0.84-18.12)	4.36 (0.74-25.78)	0.063
**Women**	**(*n* = 215)**	**(*n* = 34)**	**(*n* = 18)**	
6MWD (95% CI)	501.95 (498.73-505.16)	473.91 (464.31-483.51)[a]	436.28(419.71-452.84)[a]	0.001
**Categorical variable**				
**6MWD <500 m (n, %)**	101 (46.98)	21 (61.76)	15 (83.33)	0.004
OR (95% CI)	Reference	1.82 (0.88-3.83)	5.64 (1.59-20.06)[a]	0.002
aOR (95% CI)	Reference	2.23 (0.98-5.06)	6.19 (1.59-24.17)[a]	0.002
**6MWD <400 m (n, %)**	14 (6.51)	5 (14.71)	7 (38.89)	<0.001
OR (95% CI)	Reference	2.48 (0.83-7.38)	9.14 (3.07-27.22)[a]	<0.001
aOR (95% CI)	Reference	3.31 (0.97-11.38)	8.94 (2.18-36.71)[a]	0.002

Values of 6MWD are presented as means and 95% confidence intervals adjusted for age, body mass index, and forced expiratory volume in 1s (FEV_1) <50% of predicted. Pre-sarcopenia: pre-sarcopenia group defined according to skeletal muscle mass. Sarcopenia: sarcopenia group defined according to both skeletal muscle mass and grip strength. 6MWT, 6-min walk test; 6MWD, 6-min walk distance; OR, odds ratio; CI, confidence interval; aOR, adjusted odds ratio (OR was adjusted for age sex, body mass index, and FEV_1<50% of predicted). [a] p<0.05 compared with the normal group.
